# Increased β-Lactams dosing regimens improve clinical outcome in critically ill patients with augmented renal clearance treated for a first episode of hospital or ventilator-acquired pneumonia: a before and after study

**DOI:** 10.1186/s13054-019-2621-4

**Published:** 2019-11-27

**Authors:** Cédric Carrié, Grégoire Chadefaux, Noémie Sauvage, Hugues de Courson, Laurent Petit, Karine Nouette-Gaulain, Bruno Pereira, Matthieu Biais

**Affiliations:** 1 Anesthesiology and Critical Care Department, Hôpital Pellegrin, CHU Bordeaux, 33000 Bordeaux, France; 2 University Bordeaux Segalen, 33000 Bordeaux, France; 3 Biostatistics Unit, Délégation Recherche Clinique & Innovation, CHU de Clermont-Ferrand, Clermont-Ferrand, France

**Keywords:** Augmented renal clearance, β-Lactams, Hospital-acquired pneumonia, Ventilator-acquired pneumonia, Critical illness

## Abstract

**Background:**

Augmented renal clearance (ARC) is recognized as a leading cause of β-lactam subexposure when conventional dosing regimens are used. The main objective was to compare the clinical outcome of ARC patients treated by conventional or increased β-lactam dosing regimens for a first episode of hospital or ventilator-acquired pneumonia (HAP-VAP).

**Methods:**

In this single-center, retrospective study, every ARC patient treated by β-lactam for a first episode of HAP-VAP was included during two 15-month periods, before (*Control period*) and after (*Treatment period*) the modification of a local antibiotic therapy protocol. ARC was defined by a 24-h measured creatinine clearance ≥ 150 ml/min. The primary endpoint was defined as a therapeutic failure of the antimicrobial therapy or a HAP-VAP relapse within 28 days. Inverse probability of treatment weight (IPTW) was derived from a propensity score model. Cox proportional hazard models were used to evaluate the association between treatment period and clinical outcome.

**Results:**

During the study period, 177 patients were included (*control period*, *N* = 88; *treatment period*, *N* = 89). Therapeutic failure or HAP-VAP relapse was significantly lower in the treatment period (10 vs. 23%, *p = 0.019*). The IPTW-adjusted hazard ratio of poor clinical outcome in the treatment period was 0.35 (95% CI 0.15–0.81), *p = 0.014*. No antibiotic side effect was reported during the treatment period.

**Conclusions:**

Higher than licensed dosing regimens of β-lactams may be safe and effective in reducing the rate of therapeutic failure and HAP-VAP recurrence in critically ill augmented renal clearance (ARC) patients.

## Introduction

Critically ill patients are at risk to develop ventilator- or hospital-acquired pneumonia (HAP-VAP), which has been associated with impaired outcomes, prolonged duration of mechanical ventilation, increase in the length of stay, and the healthcare costs [1]. In this context, appropriate antibacterial exposure is essential to improve the chances of clinical success and reduce the risk for selection of drug-resistant strains [2].

According to current practice, β-lactams are the most commonly prescribed antimicrobial agents for treatment of HAP-VAP [1]. β-Lactam antibiotics exhibit time-dependent bacterial killing, such as maximum bacterial killing occurs when the free drug concentration at the site of infection persistently exceeds 4 times the minimum inhibitory concentration (MIC) of the pathogen throughout the dosing interval (100%fT_>4xMIC_) [3]. On the other hand, optimizing antimicrobial therapy remains a complex challenge given the wide and unpredictable variability of β-lactam antibiotic concentrations in critically ill patients [4].

A “one dose fits all” approach can especially be problematic in patients with augmented renal clearance (ARC), potentially leading to subtherapeutic drug exposure and higher rates of clinical failure when conventional dosing regimens are used [5–9]. In this context, several studies previously suggested that higher than licensed β-lactam dosing regimens would be necessary to improve empirical pharmacokinetic/pharmacodynamic (PK/PD) target attainment rates in patients with ARC [10, 11]. However, evidence on clinical outcome is still controversial [12, 13].

We thus hypothesized that higher than licensed dosing regimens of β-lactams may be safe and effective in reducing the rate of therapeutic failure and HAP-VAP recurrence in critically ill patients with ARC. The main objective was to compare the clinical outcome of ARC patients treated by conventional or increased β-lactam dosing regimens for a first episode of HAP-VAP.

## Methods

### Study design, population, and settings

This single-center, retrospective observational cohort study aimed to compare two 15-month periods before (*Control period*; June 2016 to August 2017) and after (*Treatment period*; November 2017 to January 2019) the modification of a local antibiotic therapy protocol in a 25-bed Surgical and Trauma Intensive Care Unit (ICU) of Bordeaux University Hospital.

During both study periods, 24-h urinary samples were collected daily from every patient and measured creatinine clearance (CL_CR_) was calculated as follows: 24-h urinary volume ×  24-h urinary creatinine / plasma creatinine, converted in ml/min. Augmented renal clearance was defined by a CL_CR_ ≥ 150 ml/min. This threshold was arbitrarily chosen to avoid empirical underexposure regardless of the type of antibiotic, dosing and modalities of administration, and without increased risk of toxicity [11, 12].

For the purpose of this study, all consecutive patients treated by β-lactam for a first episode of HAP-VAP who displayed ARC the first day of antimicrobial therapy were eligible. Patients were excluded if the HAP-VAP was not microbiologically documented (i.e., no pathogen identified by the microbiology laboratory) or if they underwent initial inappropriate antimicrobial therapy (i.e., natural or acquired resistance to the monitored β-lactam antibiotic, total antibiotic duration < 5 days or > 10 days).

Ethical approval for this analysis was obtained from the Ethics Committee of the French Society of Anesthesiology and Intensive Care (IRB number: 00010254-2018-194) which waived the need for written consent. The patients and/or next of kin were informed about the inclusion of their anonymized health data in the database, and none declined participation. Written informed consent was waived due to the observational nature of the study.

### Antibiotic therapy protocol for HAP-VAP

During both study periods, diagnosis of HAP-VAP included a clinical suspicion (≥ two criteria including fever > 38.5 °C, leukocytosis > 10^9^/l or leukopenia < 4.10^8^/l, purulent tracheobronchial secretions and a new or persistent infiltrate on chest radiography) and confirmation by a positive quantitative culture of a respiratory sample (significant threshold ≥ 10^4^ colony-forming units [CFU]/ml) for broncho-alveolar lavages [BALs], ≥ 10^6^ UFC/ml for endotracheal aspirations and ≥ 10^7^ UFC/ml for non-invasive sputum samples) [1]. Empiric treatment options for clinically suspected VAP were determined by the attending physician, with guidance from a local antibiotic therapy protocol in accordance with French recommendations (Table 1) [1]. In our local practice, therapeutic drug monitoring could only be considered for cefepime.

**Table 1:** Empirical antibiotic therapy protocol for HAP-VAP in ARC patients (CL_CR_ > 150 ml/min)

	Control period (June 2016 to November 2017) Conventional dosing regimen	Treatment period (November 2017 to January 2019) Increased dosing regimen
Early HAP-VAP (< 5 days of hospitalization) without risk factors for NF-GNB or multidrug-resistant pathogens	• Amoxicillin-clavulanic acid (2 g IV q 8 h)	• Amoxicillin-clavulanic acid (2 g IV q 6 h)
	• Cefotaxime (2 g IV q 8 h)	• Cefotaxime (2 g IV q 6 h)
	• Ceftriaxone (2 g IV once/day)	• Ceftriaxone (2 g IV q 12 h)
Late HAP-VAP (≥ 5 days of hospitalization) and/or risk factors for NF-GNB or multidrug-resistant pathogens and/or immunosuppressive disease or therapy	Broad-spectrum β-lactam:	Broad-spectrum β-lactam:
	• Piperacillin-tazobactam (16 g/day continuously after a loading dose of 4 g)	• Piperacillin-tazobactam (20 g/day continuously after a loading dose of 4 g)
	• Cefepime (6 g/day continuously after a loading dose of 2 g over 30 min)	• Cefepime (6 g/day continuously after a loading dose of 2 g over 30 mins)
	• Ceftazidime (6 g/day continuously after a loading dose of 2 g over 30 min)	• Ceftazidime (6 g/day continuously after a loading dose of 2 g over 30 min)
	• Meropenem (6 g/day continuously or 2 g q 8 h over 240 min)	• Meropenem (6 g/day continuously or 2 g q 8 h over 240 min)
If risk factors for MRSA	Gram-positive antibiotics with MRSA activity: glycopeptides (vancomycin 15 mg/kg/day continuously after a loading dose of 25 mg/kg*) or oxazolidinones (linezolid 600 mg IV twice/day)	
If septic shock or ARDS at time of HAP-VAP	Aminoglycosides or fluoroquinolones: amikacine (20–30 mg/kg*), gentamycin (7–10 mg/kg*), or levofloxacin (500 mg twice/day)	

*Glycopeptides (vancomycin) and aminoglycosides (gentamycin, amikacin) were subsequently dosed by therapeutic monitoring

Since November 2017, the protocol was modified to adjust β-lactam dosing regimens to the 24-h urinary measured CL_CR_. In accordance with previous published data, patients displaying CL_CR_ ≥ 150 ml/min received higher than licensed dosing regimens (except for meropenem, cefepime, and ceftazidime) [9–12]. Daily dosing regimens were reduced if the patient no longer experienced ARC. De-escalation of empiric antibiotic therapy was assessed whenever possible after identification of the causative microorganism and reception of the antibiotic susceptibilities. If available, the MIC provided by the local microbiology laboratory was reported. Otherwise, the MIC of the pathogen was defined by the European Committee on Antimicrobial Susceptibility Testing (EUCAST).

Standard dosing regimens of documented β-lactam antibiotic therapy were cefazolin (100 mg/kg/day continuously after a loading dose of 2 g), amoxicillin or amoxicillin/clavulanic acid (2 g q 8 h), or third generation cephalosporin (ceftriaxone [2 g q 24 h] or cefotaxime [2 g q 8 h]). In the treatment period, patients who remained in ARC benefited from increased dosing regimens of documented β-lactam antibiotic therapy (cefazolin 150 mg/kg/day, amoxicillin or amoxicillin/clavulanic acid 2 g q 6 h, ceftriaxone 2 g q 12 h, or cefotaxime 2 g q 6 h). A 7-day course was considered sufficient unless inadequate initial antibiotic therapy, bacteremia, immunosuppressive disease, or MDR pathogen.

The overall treatment of patients with HAP-VAP during the control and treatment periods was similar except for the antimicrobial dosing regimens. There were no known significant changes to our ICU protocols, ventilation or weaning procedures, or patient population during the study period.

### Study endpoints

The primary endpoint was a composite criterion defined as a poor clinical outcome of the antimicrobial therapy, including therapeutic failure and HAP-VAP recurrence within 28 days and/or end-of-hospitalization [12].

- Therapeutic failure was defined as an impaired clinical response (persistent or worsening symptoms of HAP-VAP) with the need for escalating empirical antimicrobial therapy. Inappropriate empirical treatment was not considered as therapeutic failure. De-escalation did not qualify as therapeutic failure. Superinfections due to new causative pathogens with natural resistance to the initial antimicrobial therapy were not considered as therapeutic failure.
- Recurrence was defined by a second HAP-VAP with at least one of the initial causative bacterial strains growing at a significant concentration from a second sample after completing antibiotic therapy. Tracheobronchial colonization without evidence of pulmonary infection was not considered as HAP-VAP recurrence.

The primary outcome was assessed by one of the investigators (GC) based on clinical and microbiologic data. External validation was performed independently by at least two other investigators (CC, NS) blinded to the allocated period and antibiotic dosing regimen.

The secondary endpoints were the reported antibiotic side effects during treatment (cholestasis, cytolysis, delirium, seizure, renal failure), the secondary acquisition of antimicrobial resistance within 28 days and/or end-of-hospitalization, the duration of mechanical ventilation, the length of stay in the ICU, and the status (alive or dead) at discharge.

### Statistical analysis

Preliminary data in our institution reported poor clinical outcome in more than 25% of ARC patients treated for a first episode of HAP-VAP [8, 9]. Hypothesizing a poor clinical outcome rate < 10% after increasing β-lactam dosing regimens [12], a sample of 156 patients (78 per group) was needed to confirm this hypothesis, with a 80% power and *α* = 0.05. Results are expressed as mean ± standard deviation or median (25 to 75% interquartile range) for continuous variables and as absolute or relative frequencies for categorical variables. The data distribution was analyzed by a Kolmogorov-Smirnov test. Comparisons between continuous variables were performed using the Student *t* test or the Mann–Whitney test and categorical variables were compared using the chi-square test or Fisher’s exact test as appropriate.

As not all the protocolized β-lactam agents benefited from higher dosing regimens, a propensity score analysis was performed to predict the conditional probability for an individual patient to receive an increased β-lactam dosing regimens [14]. The covariates included in the propensity score model were as follows: time of HAP-VAP occurrence, use of norepinephrine and Cl_CR_ measured both the day of HAP-VAP and the day of microbiological documentation, type of antibiotics (ceftazidime, cefepime, and/or meropenem), and antibiotic de-escalation. Inverse probability of treatment weighting (IPTW) was used for estimating the average treatment effect on time-to-event outcomes [15]. Patients receiving either ceftazidime, cefepime, or meropenem were included on the day of de-escalation to a drug with an increased dose protocol. Cox proportional hazards regression analysis was performed to estimate the relative risks (hazard ratios [HR]) of therapeutic failure or HAP-VAP relapse between both periods among the IPTW pseudo-cohort [16].

Statistical analyses were performed using XLSTAT 2015 for Windows (Addinsoft Paris, France) and Stata software (version 13; StataCorp, USA).

## Results

### Characteristics of the population

**Fig. 1:**
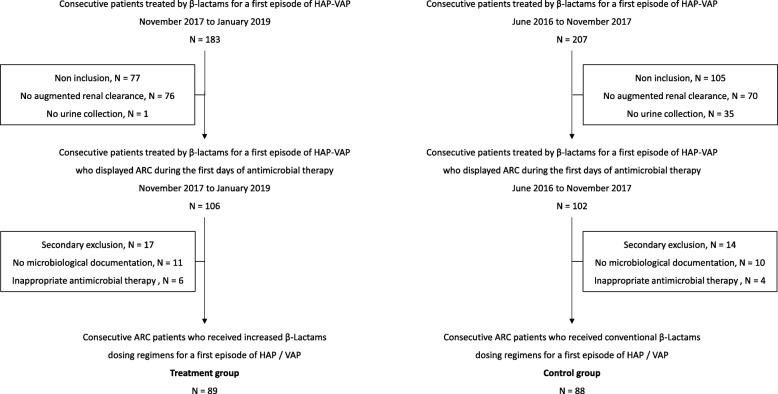
Study flow chart

During the study period, 177 patients were included in the present study (control period, *N* = 88; treatment period, *N* = 89). The study flow chart is reported in Fig. 1. Their main characteristics are shown in Table 2. Fewer patients were empirically treated by meropenem, ceftazidime, or cefepime in the treatment period (6 [7%] vs. 20 [23%], *p =* 0.003). The rate of de-escalation in these patients was 50% (13/26). Four patients (*N* = 2 in each group) died without therapeutic failure before 15 days after the termination of antimicrobial therapy. No death was related to the HAP-VAP.

**Table 2:** Characteristics of the population

Variable	Control period *N* = 88	Treatment period *N* = 89	*p*
Demographical data			
- Age (years)	45 [26–58]	45 [36–58]	*0.452*
- Male	70 (77)	77 (87)	*0.216*
- BMI (kg/m^2^)	24 [20–28]	25 [22–29]	*0.122*
Admission			
- Polytrauma	75 (80)	80 (90)	*0.347*
- Non traumatic surgery	13 (15)	9 (10)	*0.347*
SAPS 2	39 [27–48]	40 [33–49]	*0.339*
Characteristics of VAP			
- Time of occurrence (days after admission)	5 [3–8]	5 [2–7]	*0.234*
- Ventilator-acquired pneumonia	75 (85)	78 (88)	*0.639*
- Use of vasopressors	41 (46)	35 (39)	*0.329*
- PaO_2_/FiO_2_ < 200	9 (10)	7 (8)	*0.584*
- Bacteremia	3 (3)	2 (2)	*0.641*
Renal function			
- CL_CR_ the day of HAP-VAP (ml/min)	199 [170–240]	186 [160–223]	*0.013*
- CL_CR_ the day of microbiological documentation (ml/min)	191 [164–221]	174 [147–200]	*0.013*
Type of initial antimicrobial therapy			
- Piperacillin ± tazobactam	35 (40)	55 (62)	*0.003*
- Ceftriaxone or cefotaxime	16 (18)	12 (13)	*0.392*
- Ceftazidime or cefepime	14 (16)	3 (3)	*0.005*
- Amoxicillin ± clavulanic acid	9 (10)	13 (15)	*0.377*
- Cefazolin	8 (9)	3 (3)	*0.115*
- Meropenem	6 (7)	3 (3)	*0.297*
Combined antimicrobial therapy			
- Association with aminoglycosides	33 (38)	27 (30)	*0.288*
- Association with quinolones	6 (7)	1 (1)	*0.050*
- Association with Gram-positive antibiotics	10 (11)	7 (8)	*0.415*
Type of pathogen			
- Enterobacteriaceae	47 (53)	46 (52)	*0.818*
- Staphylococcus species	40 (45)	33 (37)	*0.258*
- *Haemophilus influenzae*	19 (22)	27 (30)	*0.185*
- Streptococcus/enterococcus species	11 (13)	15 (17)	*0.413*
- Non-fermenting GNB	6 (7)	11 (12)	*0.211*
- Others	1 (1)	1 (1)	*0.994*
Polymicrobial infection	29 (33)	39 (44)	*0.137*
De-escalation	26 (30)	29 (33)	*0.746*
Total duration of antimicrobial therapy (days)	7 [6–8]	7 [6–7]	*0.026*
Poor clinical outcome	20 (23)	9 (10)	*0.019*
- Therapeutic failure	7 (8)	4 (5)	*0.321*
- HAP-VAP recurrence	13 (15)	5 (6)	*0.039*
Secondary acquisition of antimicrobial resistances	4 (5)	2 (2)	*0.382*
Reported antibiotic side effects	1 (1)	0 (0)	*0.308*
MV duration (days)	12 [7–18]	10 [5–18]	*0.420*
ICU length of stay (days)	22 [15–32]	19 [14–30]	*0.216*
Death in ICU	7 (8)	8 (9)	*0.805*

Results expressed as numbers (percentage) or median [25–75 interquartile]

### Clinical outcome in the treated and control groups

Therapeutic failure or HAP-VAP relapse was reported in 9 of the 89 patients (10%) in the treatment group and 20 of the 88 patients (23%) in the control group (Table 2). No antibiotic side effect was reported in the treatment group. The median MIC values and rates of poor clinical outcome according to the initial antibiotic treatment are reported in Additional files 1, 2, and 3.

**Fig. 2:**
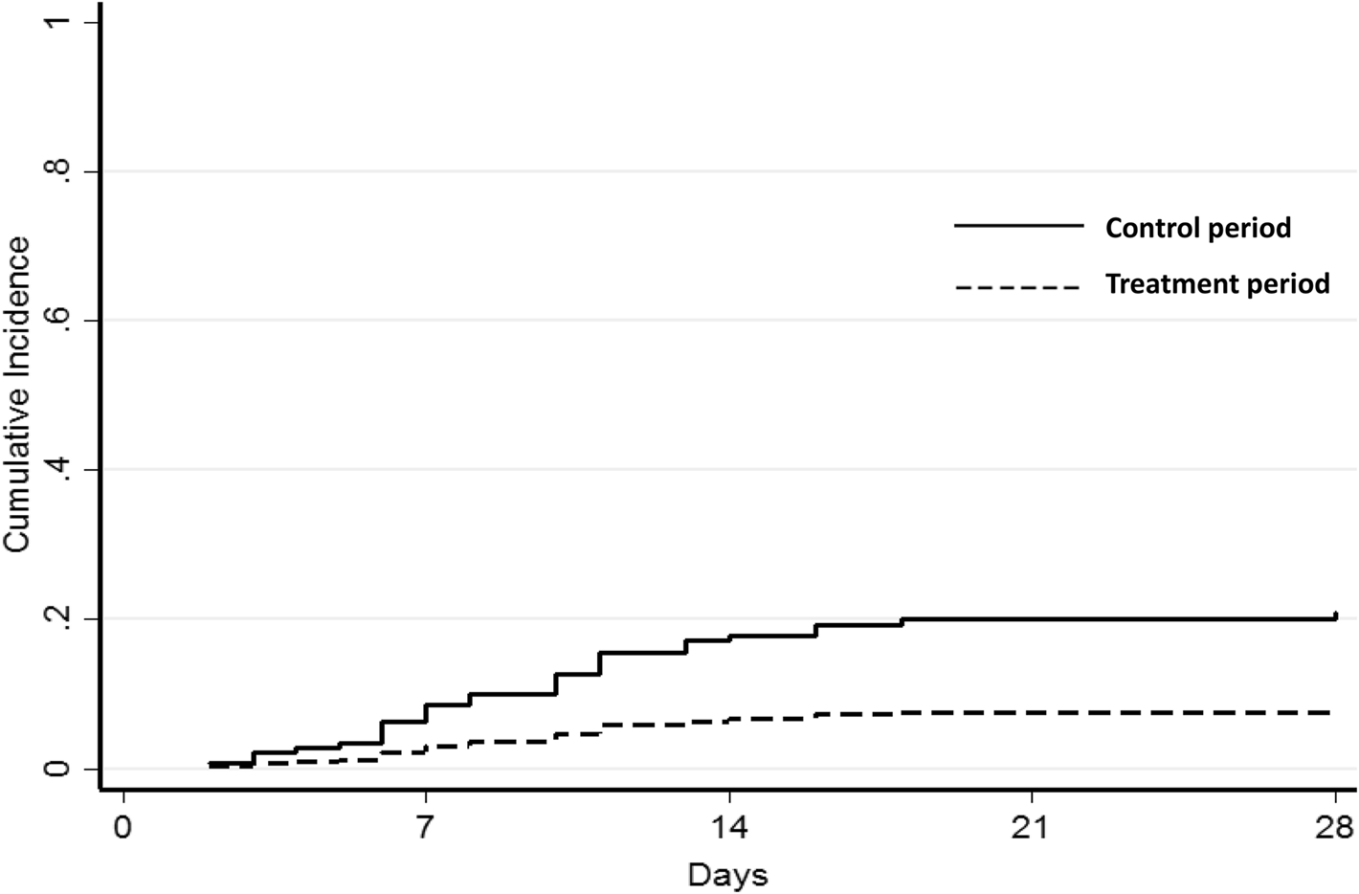
Cumulative incidence of therapeutic failure or HAP-VAP relapse among the IPTW pseudo-cohort treated by conventional (control group; *full line*) or increased (treatment group; *dotted line*) β-lactam dosing regimens. *Data adjusted on propensity score, SAPS 2 and PaO*_*2*_*/FiO*_*2*_ *ratio*

Distribution of the propensity score according to the treatment period is depicted in Additional files 1, 2, and 3. In IPTW-adjusted Cox proportional hazards regression analysis, the treatment period was associated with lower rates of therapeutic failure or HAP-VAP relapse (HR = 0.35 [95% CI 0.15–0.81], *p =* 0.014). IPTW-adjusted Kaplan–Meier curves in both treatment periods are depicted Fig. 2. The data were adjusted for propensity score, SAPS 2, and PaO_2_/FiO_2_ ratio. There was no statistical association between treatment period and secondary acquisition of resistance (HR = 0.47 [0.06–3.74], *p* = 0.47).

There was no statistical difference of MV duration, ICU length of stay, or mortality rate between the two periods. Patients experiencing therapeutic failure or HAP-VAP relapse were likely to have a longer MV duration (18 [11–24] vs. 9 [5–7], *p <* 0.0001) and ICU length of stay (29 [19–44] vs. 20 [13–30], *p =* 0.0004]), without statistical difference in the mortality rate (10% vs. 6%, *p =* 0.34).

## Discussion

Our study reports the interest of optimizing dosing strategies of antimicrobial agents in critically ill patients with augmented renal clearance. This is a major concern as inadequate antimicrobial therapy has been associated with the acquisition of antimicrobial resistance, therapeutic failure, and mortality in patients with severe sepsis or septic shock [17]. Early attention to appropriate β-lactam dosing may thus improve outcomes given the marked increase in success rates when the free concentration remains above 4 times the MIC throughout the entire dosing period (100%*f*T_>4xMIC_) [3, 9].

On the other hand, several studies have suggested a strong association between ARC, subtherapeutic β-lactam plasma concentrations, and higher rates of therapeutic failure when conventional dosing regimens are used [5–11]. ARC has been reported in approximately 16 to 100% of patients, depending on the population investigated and the definition of ARC [18]. Although most studies have defined ARC by an enhanced CL_CR_ ≥ 130 ml/min/1.73m^2^, we arbitrarily chose a cut-off of ≥ 150 ml/min for increasing β-lactam dosing regimens. Indeed, the threshold of CL_CR_ defining ARC should rely on the inherent risk of antibiotic underexposure depending of the type of β-lactam, dosing regimen, and modalities of administration. For β-lactams administered by continuous infusion, a previous study suggested that no underdosing was observed for CL_CR_ ≤ 170 ml/min [9]. Thresholds higher than 130 ml/min have also been reported for β-lactams administered by intermittent infusion (150 ml/min for ceftriaxone, up to 190 ml/min for amoxicillin-clavulanic acid) [11, 19]. Interestingly, the same threshold was also suggested by Roberts et al., the vast majority of patients with CL_CR_ values > 150–160 ml/min requiring a dose increase to reach trough or steady-state concentrations at four times MIC of the known pathogen [20].

Our antibiotic therapy protocol was thus in accordance with previous studies which suggested that higher than licensed dosing regimens would be necessary in patients with ARC. Piperacillin-tazobactam has been the most studied antibiotic in this context, where previous studies demonstrated that standard intermittent dosing regimens were unlikely to achieve optimal antibiotic exposure [21–23]. Although other studies suggested a significant improvement of PK/PD target attainment rates when extended or continuous infusions are used, patients with CL_CR_ ≥ 150–170 ml/min remained at risk for empirical underexposure [9, 10]. In this context, higher than licensed doses of piperacillin-tazobactam (20 g/2.5 g/day by continuous infusion) allowed attaining the empirical PK/PD target in 100% patients with CL_CR_ ≥ 150 ml/min, without excessive dosing [12].

Data on other β-lactams in patients with ARC are scarce. For ceftriaxone, we previously demonstrated high rates of empirical target underdosing in patients with CL_CR_ ≥ 150 ml/min. In this context, Monte Carlo simulations suggested a dose of 2 g twice a day to achieve a 100% *fT*_>MIC_ when targeting a theoretical MIC at the upper limit of the susceptibility range [11]. Dosing simulations for amoxicillin-clavulanic acid also supported the use of increased dosing regimens (2 g four times daily) for ARC patients when using a target MIC of 8 mg/l and a pharmacodynamic target of 50% fT_>MIC_, with little accumulation of clavulanic acid [21, 24]. Considering cefazolin, only an ancillary pilot study suggested a higher rate of underdosing in ARC patients, especially when PK/PD targets were adjusted to the inoculum effect [25].

Finally, some β-lactam antibiotics were not adjusted in the treatment period as no data had previously demonstrated that ARC patients may benefit for enhanced dosing regimen of meropenem, ceftazidime, or cefepime. Concerning meropenem, various Monte Carlo simulation studies suggested that 2000 mg q8h either by extended (180-min infusion) or continuous infusion allowed achieving PK/PD targets even with CL_CR_ of 130–250 ml/min [26]. In a former study, ARC (CL_CR_ ≥ 170 ml/min) was not associated with an increased rate of subexposure (fT_<4xMIC_) for those antibiotic agents administered 6 g/day continuously [9]. However, this study was impaired by a small sample size of ARC patients treated by ceftazidime or cefepime and did not allow any conclusion about the optimal dosing regimen in ARC patients treated by ceftazidime and cefepime for less-susceptible pathogens. This reason might explain the observed difference concerning the type of initial broad-spectrum antimicrobial therapy (ceftazidime or cefepime and/or piperacillin ± tazobactam) between both periods in the present study. Patients empirically treated by meropenem, ceftazidime, or cefepime were not excluded for the analysis as most of them underwent antibiotic de-escalation that could require dose-adjustment in the *treatment* period.

A prompt recognition of ARC is thus of paramount importance for optimizing empirical antibiotic dosing regimens. However, commonly used formulas frequently misclassify ARC and may underestimate the risk of β-lactam underdosing [27, 28]. Although several screening tools have shown adequate predictive abilities for identifying patients with ARC, 24-h measured CL_CR_ must remain the reference and should be monitored daily in at-risk patients [29, 30]. On the other hand, dose adaptations solely based on CL_CR_ are probably not sufficient to achieve target therapeutic concentrations [31]. The relationship between CL_CR_ and drug concentrations is probably far more complex and dose-adaptions are probably best guided by a validated Population PK model and not by CL_CR_ alone [32]. Moreover, a special attention should be granted to detect dose-dependent neurotoxicity, especially when using higher than licensed doses of β-lactams [33]. Intra-patient variability of CL_CR_ and drug concentrations over time must require subsequent dose adjustment guided by CL_CR_ values, pathogen susceptibility, and therapeutic drug monitoring [34]. However, the safety of increased dosing regimens in ARC patients has been reported in previous studies, all the reported concentrations being significantly under the supposed toxic cutoff [12, 13]. Currently, therapeutic drug monitoring is required to adjust daily regimens in critically ill patients.

Several limitations should be considered. First, the main limitation relied on the retrospective before–after design that could lead to selection and interpretation bias. Even if there were no known significant changes to our local procedures, our results may be driven by the preferential use of increased dosing regimens of piperacillin-tazobactam. However, the propensity score matching allowed reducing the effects of confounding covariates directly influencing the choice of probabilistic or documented antibiotic therapy. Furthermore, we aimed to focus on HAP-VAP as they meet widely used criteria for diagnostic and management, where therapeutic failure is not related to inadequate surgical source control. Second, the rate of antibiotic side effects is very limited, although probably related to an underreporting in the medical records. Finally, the study design did not allow adequate MIC determination and therapeutic drug monitoring. Only pharmacological dosing could confirm the association between higher rates of PK/PD target attainment and lower rates of therapeutic failure in critically ARC patients treated by increased β-lactam dosing regimens.

## Conclusion

Higher than licensed dosing regimens of β-lactams may be safe and effective in reducing the rate of therapeutic failure and HAP-VAP recurrence in critically ill ARC patients treated for a first episode of HAP-VAP.

## Supplementary information

**Additional file 1.** Distribution of therapeutic failure rates according to initial antibiotic treatment.

**Additional file 2.** Median values of MIC according to initial antibiotic treatment.

**Additional file 3.** Distribution of propensity score between treatment and control groups.

## Data Availability

The datasets used and/or analyzed during the current study are available from the corresponding author on reasonable request.

## Abbreviations

ARC: Augmented renal clearance
CL_Cr_: Measured creatinine clearance
EUCAST: European Committee on Antimicrobial Susceptibility Testing
HAP: Hospital-acquired pneumonia
HR: Hazard ratio
ICU: Intensive care unit
MIC: Minimum inhibitory concentration
OR: Odds ratio
PD: Pharmacodynamic
PK: Pharmacokinetic
MV: Mechanical ventilation
VAP: Ventilator-acquired pneumonia

## References

[1] Leone M, Bouadma L, Bouhemad B (2018). Hospital-acquired pneumonia in ICU. Anaesth Crit Care Pain Med..

[2] Taccone FS, Laupland KB, Montravers P (2016). Continuous infusion of β-lactam antibiotics for all critically ill patients?. Intensive Care Med.

[3] Mc Kinnon PS, Paladino JA, Schentag JJ (2008). Evaluation of area under the inhibitory curve (AUIC) and time above the minimum inhibitory concentration (T>MIC) as predcitors of outcome for cefepime and ceftazidime in serious bacterial infections. Int J Antimicrob Agents.

[4] Roberts JA, Paul SK, Akova M (2014). DALI: defining antibiotic levels in intensive care unit patients: are current β-lactam antibiotic doses sufficient for critically ill patients?. Clin Infect Dis.

[5] Udy AA, Varghese JM, Altukroni M (2012). Subtherapeutic initial β-lactam concentrations in select critically ill patients: association between augmented renal clearance and low trough drug concentrations. Chest..

[6] Huttner A, Von Dach E, Renzoni A (2015). Augmented renal clearance, low β-lactam concentrations and clinical outcomes in the critically ill: an observational prospective cohort study. Int J Antimicrob Agents.

[7] Claus BO, Hoste EA, Colpaert K, Robays H, Decruyenaere J, De Waele JJ (2013). Augmented renal clearance is a common finding with worse clinical outcome in critically ill patients receiving antimicrobial therapy. J Crit Care.

[8] Carrie C, Bentejac M, Cottenceau V (2018). Association between augmented renal clearance and clinical failure of antibiotic treatment in brain-injured patients with ventilator-acquired pneumonia: a preliminary study. Anaesth Crit Care Pain Med..

[9] Carrie C, Petit L, d’Houdain N (2018). Association between augmented renal clearance, antibiotic exposure and clinical outcome in critically ill patients receiving high doses of β-lactams administered by continuous infusion: a prospective observational study. Int J Antimicrob Agents.

[10] Carrié C, Legeron R, Petit L (2018). Higher than standard dosing regimen are needed to achieve optimal antibiotic exposure in critically ill patients with augmented renal clearance receiving piperacillin-tazobactam administered by continuous infusion. J Crit Care.

[11] Ollivier J, Carrié C, d'Houdain N, et al. Are standard dosing regimens of ceftriaxone adapted for critically ill patients with augmented creatinine clearance? A prospective observational study. Antimicrob Agents Chemother. 2019;63(3):e02134-18. 10.1128/AAC.02134-18 PMC6395919 30602511

[12] Besnard T, Carrié C, Petit L, Biais M (2019). Increased dosing regimens of piperacillin-tazobactam are needed to avoid subtherapeutic exposure in critically ill patients with augmented renal clearance. Crit Care.

[13] Dhaese SAM, Roberts JA, Carlier M, Verstraete AG, Stove V, De Waele JJ (2018). Population pharmacokinetics of continuous infusion of piperacillin in critically ill patients. Int J Antimicrob Agents.

[14] Austin PC (2011). An introduction to propensity score methods for reducing the effects of confounding in observational studies. Multivar Behav Res.

[15] Austin PC, Stuart EA (2015). Moving towards best practice when using inverse probability of treatment weighting (IPTW) using the propensity score to estimate causal treatment effects in observational studies. Stat Med.

[16] Austin PC, Schuster T, Platt RW (2015). Statistical power in parallel group point exposure studies with time-to-event outcomes: an empirical comparison of the performance of randomized controlled trials and the inverse probability of treatment weighting (IPTW) approach. BMC Med Res Methodol.

[17] Rhodes A, Evans LE, Alhazzani W (2017). Surviving sepsis campaign: international guidelines for management of sepsis and septic shock: 2016. Intensive Care Med.

[18] Sime FB, Udy AA, Roberts JA (2015). Augmented renal clearance in critically ill patients: etiology, definition and implications for beta-lactam dose optimization. Curr Opin Pharmacol.

[19] Carlier M, Noë M, De Waele JJ (2013). Population pharmacokinetics and dosing simulations of amoxicillin/clavulanic acid in critically ill patients. J Antimicrob Chemother.

[20] Roberts JA, Ulldemolins M, Roberts MS (2010). Therapeutic drug monitoring of beta-lactams in critically ill patients: proof of concept. Int J Antimicrob Agents.

[21] Carlier M, Carrette S, Roberts JA (2013). Meropenem and piperacillin/tazobactam prescribing in critically ill patients: does augmented renal clearance affect pharmacokinetic/pharmacodynamic target attainment when extended infusions are used?. Crit Care.

[22] Andersen MG, Thorsted A, Storgaard M, Kristoffersson AN, Friberg LE, Öbrink-Hansen K (2018). Population pharmacokinetics of piperacillin in sepsis patients: should alternative dosing strategies be considered?. Antimicrob Agents Chemother.

[23] Udy AA, Lipman J, Jarrett P (2015). Are standard doses of piperacillin sufficient for critically ill patients with augmented creatinine clearance?. Crit Care.

[24] Haeseker M, Havenith T, Stolk L, Neef C, Bruggeman C, Verbon A (2014). Is the standard dose of amoxicillin-clavulanic acid sufficient?. BMC Pharmacol Toxicol.

[25] Petit L, Carrié C, Hisz Q, d’Houdain N, Breilh D, Sztark F (2018). Are standard doses of cefazolin adapted for methicillin-susceptible *Staphylococcus aureus* respiratory infections in critically ill patients with augmented renal clearance?. Ann Intensive Care.

[26] Tamatsukuri T, Ohbayashi M, Kohyama N (2018). The exploration of population pharmacokinetic model for meropenem in augmented renal clearance and investigation of optimum setting of dose. J Infect Chemother.

[27] Baptista JP, Udy AA, Sousa E (2011). A comparison of estimates of glomerular filtration in critically ill patients with augmented renal clearance. Crit Care.

[28] Carrié C, Rubin S, Sioniac P, Breilh D, Biais M (2018). The kinetic glomerular filtration rate is not interchangeable with measured creatinine clearance for prediction of piperacillin underexposure in critically ill patients with augmented renal clearance. Crit Care.

[29] Barletta JF, Mangram AJ, Byrne M (2017). Identifying augmented renal clearance in trauma patients: validation of the augmented renal clearance in trauma intensive care scoring system. J Trauma Acute Care Surg.

[30] Carrie Cedric, Lannou Alexandre, Rubin Sebastien, De Courson Hugues, Petit Laurent, Biais Matthieu (2019). Augmented renal clearance in critically ill trauma patients: A pathophysiologic approach using renal vascular index. Anaesthesia Critical Care & Pain Medicine.

[31] Jacobs A, Taccone FS, Roberts JA, Jacobs F, Cotton F, Wolff F, et al. β-Lactam dosage regimens in septic patients with augmented renal clearance. Antimicrob Agents Chemother. 2018;62(9). 10.1128/AAC.02534-17 PMC6125556 29987138

[32] Felton TW, Roberts JA, Lodise TP, Van Guilder M, Boseli E, Neely MN (2018). Individualization of piperacillin dosing for critically ill patients: dosing software to optimize antimicrobial therapy. Antimicrob Agents Chemother.

[33] Beumier M, Casu GS, Hites M, Wolff F, Cotton F, Vincent JL (2015). Elevated β-lactam concentrations associated with neurological deterioration in ICU septic patients. Minerva Anestesiol.

[34] Imani S, Buscher H, Marriott D, Gentili S, SAndaradura I (2017). Too much of a good thing: a retrospective study of β-lactam concentration-toxicity relationships. J Antimicrob Chemother.

