# Supplementary material for: Increased β-Lactams dosing regimens improve clinical outcome in critically ill patients with augmented renal clearance treated for a first episode of hospital or ventilator-acquired pneumonia: a before and after study
Source: Crit Care. 2019 Nov 27;23:379. doi: 10.1186/s13054-019-2621-4 (PMC6881978; doi:10.1186/s13054-019-2621-4)
Supplement: Supplementary file 1 — Additional file 1. Distribution of therapeutic failure rates according to initial antibiotic treatment. [file 13054_2019_2621_MOESM1_ESM.pdf]

Supplementary Data. Distribution of therapeutic failure rates according to initial antibiotic treatment.

Results expressed as percentages [95% confidence interval]

	**Control period**<br>**N = 88**	**Treatment period**<br>**N = 89**	**p**
**Piperacillin ± Tazobactam**<br>*N = 90*	17 [5 – 30]	6 [0 – 13]	0.12
**Ceftriaxone or cefotaxime**<br>*N = 28*	31 [9 – 54]	25 [1 – 50]	0.72
**Amoxicillin ± Clavulanic Acid**<br>*N = 22*	22 [0 – 49]	15 [0 – 35]	0.68
**Ceftazidime or Cefepime**<br>*N = 17*	21 [0 – 43]	0 [0 – 0]	0.38
**Cefazolin**<br>*N = 11*	38 [4 – 71]	0 [0 – 0]	0.21
**Meropenem**<br>*N = 9*	17 [0 – 46]	33 [0 – 87]	0.57
