# Supplementary material for: Increased β-Lactams dosing regimens improve clinical outcome in critically ill patients with augmented renal clearance treated for a first episode of hospital or ventilator-acquired pneumonia: a before and after study
Source: Crit Care. 2019 Nov 27;23:379. doi: 10.1186/s13054-019-2621-4 (PMC6881978; doi:10.1186/s13054-019-2621-4)
Supplement: Supplementary file 2 — Additional file 2. Median values of MIC according to initial antibiotic treatment. [file 13054_2019_2621_MOESM2_ESM.pdf]

Supplementary Data. Median values of MIC according to initial antibiotic treatment. *The MIC values were provided by the local microbiology laboratory in 30 patients (17%). Results expressed as median [25 – 75 interquartile]*

	**Control period** N = 88	**Treatment period** N = 89	***p***
**Piperacillin ± Tazobactam** *N = 90*	4 [2.5 – 4]	4 [4 – 4]	*0.01*
**Ceftriaxone or cefotaxime** *N = 28*	0.5 [0.4 – 0.6]	0.5 [0.1 – 0.9]	*0.51*
**Amoxicillin ± Clavulanic Acid** *N = 22*	2 [1.8 – 3.5]	2 [0.5 – 2]	*0.36*
**Ceftazidime or Cefepime** *N = 17*	1 [0.5 – 1]	4 [3 - 6]	*0.02*
**Cefazolin** *N = 11*	2 [2 – 2]	2 [1.3 – 2]	*0.65*
**Meropenem** *N = 9*	0.4 [0.3 – 0.5]	0.1 [0.1 – 0.1]	*0.14*
