# Supplementary figures and images for: Increased β-Lactams dosing regimens improve clinical outcome in critically ill patients with augmented renal clearance treated for a first episode of hospital or ventilator-acquired pneumonia: a before and after study
Source: Crit Care. 2019 Nov 27;23:379. doi: 10.1186/s13054-019-2621-4 (PMC6881978; doi:10.1186/s13054-019-2621-4)

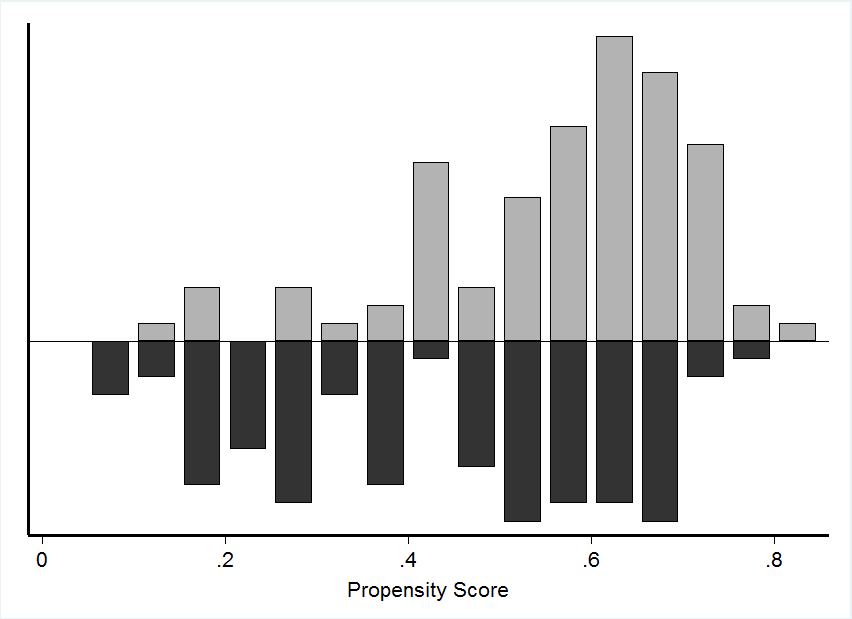

Supplement: Supplementary file 3 — Additional file 3. Distribution of propensity score between treatment and control groups. [file 13054_2019_2621_MOESM3_ESM.tiff]
